# The Possible Role of Sphincteroplasty and Surgical Sphincterotomy in the Pathogenesis of Recurrent Common Duct Brown Stones

**DOI:** 10.1155/1991/89069

**Published:** 1991

**Authors:** Francesco Cetta

**Affiliations:** 1 Institute of Surgical Pathology University of Siena Italy; 2 Instituto di Patologia Chirurgica Nuovo Policlinico Siena 53100 Italy

## Abstract

The hypothesis has been tested that postcholecystectomy common duct stones of the brown subtype are a consequence of three factors: bile infection, old age and previous sphincterotomy. It was found that: (i) 27 of 39 consecutive patients with recurrent common duct stones had brown stones. Nineteen of these 27 patients (70.3%) had previous sphincterotomy or sphincteroplasty: (ii) six of 15 patients with stone and bile analysis both at the time of cholecystectomy and at the second operation and who had sterile operative bile and non brown stones at the first operation, formed brown stones after T-tube drainage and after the onset of bile infection; (iii) patients with both intra and postoperative negative bile culture (n = 39 out of 137) had a lower mean age (50.5 years) and less frequently had a sphincterotomy than did individuals with a negative culture at operation, who subsequently had bile infection (n = 37; mean age 58.5 years; sphincterotomy in 88.8% of cases).

In addition, in a follow up study of 105 patients with sphincterotomy and with sphincteroplasty (including ERCP or i.v. cholangiography in all cases), mean follow-up interval 6.1 years, 11.3 % of patients had brown recurrent common duct stones.

It is suggested that, since brown recurrent common duct stones are secondary to bile stasis and infection and the duodenum is going to be colonized by bacteria with increasing age, sphincterotomy (and subsequent stricture), facilitating bile contamination and bacterial overgrowth, could be one of the major determinants of brown recurrent common duct stones (RCS) formation. In particular, more than 11% of the patients with a sphincterotomy are going to form in the future RCS of the brown subtype.


*HPB Surgery*, 1991, Vol. 4, pp 261–270
Reprints available directly from the publisher
Photocopying permitted by license only

© 1991 Harwood Academic Publishers GmbH
Printed in the United Kingdom

### THE POSSIBLE ROLE OF SPHINCTEROPLASTY AND SURGICAL SPHINCTEROTOMY IN THE PATHOGENESIS OF RECURRENT COMMON DUCT BROWN STONES

FRANCESCO CETTA
*Institute of Surgical Pathology, University of Siena, Italy*

*(Received 12 February 1991)*

The hypothesis has been tested that postcholecystectomy common duct stones of the brown subtype are a consequence of three factors: bile infection, old age and previous sphincterotomy. It was found that: (i) 27 of 39 consecutive patients with recurrent common duct stones had brown stones. Nineteen of these 27 patients (70.3%) had previous sphincterotomy or sphincteroplasty: (ii) six of 15 patients with stone and bile analysis both at the time of cholecystectomy and at the second operation and who had sterile operative bile and non brown stones at the first operation, formed brown stones after T-tube drainage and after the onset of bile infection; (iii) patients with both intra and postoperative negative bile culture (n = 39 out of 137) had a lower mean age (50.5 years) and less frequently had a sphincterotomy than did individuals with a negative culture at operation, who subsequently had bile infection (n = 37; mean age 58.5 years; sphincterotomy in 88.8% of cases).

In addition, in a follow up study of 105 patients with sphincterotomy and with sphincteroplasty (including ERCP or i.v. cholangiography in all cases), mean follow-up interval 6.1 years, 11.3 % of patients had brown recurrent common duct stones.

It is suggested that, since brown recurrent common duct stones are secondary to bile stasis and infection and the duodenum is going to be colonized by bacteria with increasing age, sphincterotomy (and subsequent stricture), facilitating bile contamination and bacterial overgrowth, could be one of the major determinants of brown recurrent common duct stones (RCS) formation. In particular, more than 11% of the patients with a sphincterotomy are going to form in the future RCS of the brown subtype.

KEY WORDS: Sphincterotomy, bile stasis and infection, common duct brown stones

Sphincterotomy is a common surgical and endoscopic procedure with an increased range of indications [1–5], both in the treatment of papillary stenosis and of common duct stones. However, even if sphincterotomy and sphincteroplasty are widespread procedures, with a low perioperative mortality and morbidity, very little is known on their possible long term side effects [1–6].

In particular, the incidence of recurrent duct stones after damage of the sphincteric mechanism has never been evaluated carefully.

We have recently documented that[7]:

1) Recurrent common duct stones of the brown subtype are usually associated with infection by E. coli; 2) Bile infection is not secondary to the presence of brown stones, but precedes brown stones formation; 3) Bile infection by E. coli is, together with bile stasis, the main factor in the pathogenesis of brown stones.

---

Correspondence to: Prof. Francesco Cetta, Instituto di Patologia Chirurgica, Nuovo Policlinico, 53100 Siena, Italy

The aim of the present study has been to evaluate the possible role of sphinctero-tomy and sphincteroplasty (and of biliary enteric anastomosis) in the pathogenesis of recurrent common duct brown stones.
The following features were specifically evaluated:

(i) the prevalence of brown stones among recurrent common duct stones;
(ii) the prevalence of sphincterotomy in patients with brown recurrent common duct stones;
(iii) the incidence of brown recurrent common duct stones in a follow-up study of patients with surgical sphincterotomy and sphincteroplasty;
(iv) the incidence of sphincterotomy and/or bile infection in patients with non brown postcholecystectomy stones.
(v) the incidence of postoperative positive bile culture in patients with sphinctero-tomy and previous negative bile culture. The latter study was specifically performed to assess the importance of the loss of the sphincteric mechanism in the pathogene-sis of bactibilia in patients with a non sterile duodenum (i.e. the great majority of old patients);
(vi) in particular, the following hypothesis has been tested, namely that recurrent common duct stones of the brown subtype, which are the most frequent, are a consequence of three factors: bile infection, age and previous sphincterotomy.

### MATERIAL AND METHODS

### *Patient Groups*

In order to test the hypothesis presented in the introduction, four different groups of individuals belonging to different patient populations have been evaluated.

1. *51 patients with postcholecystectomy stones* (found in a surgical series of 1000 consecutive patients who underwent surgery for biliary tract diseases in our institution from 1980 to 1988 and who had systematic stone and bile analysis in every case). Thirty-nine of these 51 patients were considered as having true recurrent common duct stones (i.e. entirely formed in the common duct after cholecystectomy[7-13].

These patients were selected in order to: a) evaluate the prevalence of stones of the brown subtype among postcholecystectomy stones; b) document the frequency of association between brown recurrent common duct stones and sphincterotomy or sphincteroplasty.

2. *15 patients* belonging to the group of 51 postcholecystectomy stone subjects, *who had stone and bile analysis not only at the second operation* (i.e. when they had postcholecystectomy stones), *but also at cholecystectomy time* (i.e. they were included twice in the prospective study, at cholecystectomy and at the second operation).

This special group of patients (the largest in the literature) was specifically analyzed in order to demonstrate: a) that brown stones can form in postcholecys-tectomy patients with previous non brown stones; b) the relative importance of bile contamination in their formation.

3. *131 patients*, also belonging to the same group of 1000 patients, *who had T-tube*

*drainage and monitoring of operative and postoperative bile bacteriology through the T-tube.* This group of patients (some with and some without associated sphincterotomy) was analyzed in order to evaluate the relative importance of the T-tube, age and sphincterotomy in the postoperative contamination of previously sterile bile.

4. *287 patients, who underwent sphincterotomy and sphincteroplasty from 1960 to 1984* (therefore only partly belonging to the previous group of 1000 patients) and *who were followed-up in the same period.*

This study was undertaken to document the incidence of postsphincterotomy stones and in particular the incidence of brown stones at various intervals post sphincterotomy. All patients with proven recurrent stones in this group underwent surgery for stone removal.

### METHODS

In all of the 1000 consecutive patients, clinical and laboratory findings, gallbladder histology, bile culture and bile pH were related to the analysis of stone composition by X-ray diffractometry and infrared spectroscopy. A detailed description of techniques has already been reported[7–13]

In particular, bile samples for bile culture were always obtained at operation and, in patients with a T-tube (n = 131), every two days in the postoperative period. Bile culture was considered positive when a concentration greater than $10^6$-Colony Forming Units (CFU)/ml. was found. Details of these methods have been reported elsewhere[7–13].

Data concerning stone composition and bile analysis in the entire group of 1000 patients have been reported elsewhere[15].

Sphincterotomy was usually performed by a 8–12 mm incision of the sphincter of Oddi, even if sometimes a longer incision was necessary to remove stones of greater diameter. With the help of a transpapillary probe, introduced through the common duct, a small duodenotomy was performed (about 10 mm in length). The papilla was exposed and incised towards the right of the midline in a 45° angle between cephalad and strictly lateral. Primary suture of the common duct (because of the high incidence of clinical cholangitis or bile infection at operation) was performed in less than 10% of patients with choledochotomy. A T-tube was usually inserted and removed 10–12 days postoperatively, after performing control cholangiography.

Sphincteroplasty was performed as a routine procedure by another surgical team in 133 cases from 1960 to 1984. In addition to the incision of the sphincter of Oddi, according to the above mentioned indications, it also included the insertion of 2–3 absorbable fine sutures, approximating the choledochal and duodenal mucosa, mainly on the lateral side after identification of the pancreatic duct.

Among the 287 patients with sphincterotomy or sphincteroplasty from 1960 to 1984 who were followed-up, a complete study including radiological review, was performed in 61 (out 154) patients with sphincterotomy (24 males, 37 females, mean age = 64.9) and 44 (out of 133) with sphincteroplasty (15 males, 29 females, mean age = 65.5). All of these patients (n = 105), who had routine cholangiography at cholecystectomy, underwent i.v. cholangiography and/or endoscopic retrograde cholangio pancreatography (ERCP) at various intervals postoperation. In particular, 47 patients had i.v. cholangiography, 19 had ERCP and 39 both procedures. The mean follow-up interval was 6.7±2.5 years in patients with sphincterotomy and 5.4±3 years in patients with sphincteroplasty.

Statistical analysis : comparison between means were made using Student's t-test and between proportions using X square analysis.

### RESULTS

### *1. Patients with Postcholecystectomy Stones (n = 51)*

Age, sex, bile culture and type of stones in this group are shown in Table 1. *Twenty-seven patients* (11 males, 16 females, mean age 69.5) *had typical recurrent brown stones.* They were single in 10 cases and multiple in 17 cases. Brown stones were soft in consistence, easily crushed with fingers. In cross section they usually showed alternate light and tan layers, but in some cases they were true aggregates of brown mud, with no typical structure. Brown stones contained non absorbable suture material in 5 cases and phytobezoars in other 5 cases. Recurrent brown stones always had the same structure and composition both in the centre and in the periphery, never presenting a central nucleus different from the rim. Bile culture was always positive. In particular, E. coli was always found in a concentration greater than $10^6$ CFU/ml., alone or in association with other bacteria (Proteus, Pseudomonas, Klebsiella, Enterococcus) in all of the patients with brown stones.

**Table1** Data in patients with postcholecystectomy stones (n = 51 of 1000 consecutive surgical patients.

| | *N* | *Age* | *M* | *F* | *Bile culture+* |
|---|---|---|---|---|---|
| Recurrent brown stones | 27 | 69.5 | 11 | 16 | 27 |
| suture material ↑ | 5 | 50.2 | 1 | 4 | 3 |
| Recurrent non brown stones | | | | | |
| ↓ long cystic remnant | 7 | 65.5 | 2 | 5 | 3 |
| Retained stones | 12 | 52.2 | 7 | 5 | 6 |
| Total | 51 | 63.5 | 21 | 30 | 39 |

*Twelve patients* (7 males, 5 females, mean age 52.2) *had retained stones.* They were similar to stones removed at cholecystectomy in 4 cases, while they had a central nidus similar to gallbladder stones and a laminate brown periphery (in 6 cases), or were associated with entirely brown stones. Bile culture was positive in 6 cases (50%).

*Twelve patients had non brown postcholecystectomy stones* which could be considered as formed entirely postcholecystectomy. *Six of these* patients *had cholesterol stones associated with a long cystic remnant.* Therefore they could have formed postcholecystectomy, but not primarily in the common duct. They were not considered retained, because in 5 cases patients had a postoperative asymptomatic interval before stone recurrence greater than 10 years and in the last case (see following paragraph) we could document the postcholecystectomy formation of the cholesterol stone. On the contrary, *5 patients* with no cystic remnant *had non brown stones, containing non absorbable suture material.* (Stones primarily formed in the common duct). The last patient had a long cystic remnant in association with stones containing suture material.

The surgical procedures that were associated with cholecystectomy at the first operation are reported in Table 2 (brown recurrent common duct stones) and Table 3 (non brown recurrent common duct stones and RTS). Only 14 of the 27 patients with brown recurrent common duct stones had had the first operation in our Institution. In these cases surgical sphincterotomy was performed according to previously reported criteria. In particular, (Table 2) 70.3% of patients with recurrent common duct stones had a previous operation on the papilla of Vater. On the contrary, only one of the 24 patients with non brown recurrent common duct stones or RTS (4.2%) had a previous sphincterotomy ($p < 0.001$).

In addition, by comparing Table 2 and 3, it is evident that bile infection was less frequent in the second group (44–60% versus 100% of the group with brown recurrent common duct stones). Mean age was also lower in the group with non brown stones. In particular, age difference between the group with brown recurrent common duct stones and the small subgroup of patients with non brown recurrent common duct stones containing suture material (69.5 versus 50.2) or the subgroup with retained stones (69.5 versus 52.2) resulted as highly significant ($p < 0.001$).

Recurrent brown stones formed early enough after operation in half of the patients. In particular, in 13 of 27 patients (48%), stones reformed within 4 years. In 4 patients, stones reformed between 4 and 6 years and in 3 patients between 6 and 12 years. In 7 of 27 patients (26%) stones were removed after 12 years (12–27 years).

### 2. *Patients with Stone and Bile Analysis both at Cholecystectomy and at the Second Operation (n = 15)*

Nine of the 15 patients who had stone and bile analysis both at cholecystectomy and at the second operation had brown recurrent common duct stones. None of them had common duct brown stones at the cholecystectomy time. Eight had sphincterotomy, while one had choledochotomy plus T tube drainage. Bile culture was already positive at the first operation in 3 patients. In the other 6 patients bile culture was negative at the cholecystectomy time and became positive for E. coli (> CFU /ml.) 8–14 days postoperatively (as detected by T-tube bile cultures). Typical brown stones, with no nucleus different from the periphery were found at reoperation. Bile culture was still positive for E. coli. Stones at the cholecystectomy time had been cholesterol in 5 cases and black pigment in the last case.

The remaining 6 of the 15 patients had non brown stones at the reoperation. All of these patients had cholesterol or mixed stones at the first operation. Stones were retained in 3 cases, (bile culture positive in 2 cases, one having concomitantly cholesterol and brown stones), non brown recurrent common duct stones containing suture material in 2 cases (bile culture positive in one case), non brown recurrent common duct stones associated with a long cystic remnant in the last case.

**Table 2** Previous operation associated with cholecystectomy in patients with recurrent common duct brown stones (n = 27; mean age 69.5; positive culture 100%).

| | *N* | *%* |
|---|---|---|
| Sphincterotomy or Sphincteroplasty | 19 | 70.3 |
| Choledochoduodenostomy | 3 | 11.1 |
| Hepaticojejunostomy | 1 | 3.7 |
| Choledochotomy | 4 | 14.8 |
| Total | 27 | 100 |

**Table 3** Previous operations associated with cholecystectomy in patients with non brown recurrent stones containing suture material (SM) (mean age 50.2, positive culture 60%) or associated with long cystic remnant (CR) (mean age 62.5, positive culture 44%) and in patients with retained stones (RTS) (mean age 52.2, positive culture 50%).

| | *SM* | | *CR* | | *RTS* | | *Total* |
|---|---|---|---|---|---|---|---|
| | N | % | N | % | N | % | |
| SPHT or SPHP | – | – | – | – | 1 | 8.3 | 1 |
| Choledochoduodenotomy | – | – | – | – | – | – | – |
| Hepaticojejunostomy | – | – | – | – | – | – | – |
| Choledochotomy | 2 | 40 | 2 | 28.6 | 3 | 25 | 7 |
| No common duct procedure | 3 | 60 | 5 | 71.4 | 8 | 66.6 | 16 |
| Total | 5 | 100 | 7 | 100 | 12 | 100 | 24 |

SPHT = Sphincterotomy; SPHP = Sphincteroplasty

In particular, in a 40-year-old female, a large cholesterol stone containing non absorbable suture material was found. This patient had a choledochotomy and T-tube drainage (but not sphincterotomy) 4 years before. At the second operation, the duct was greatly dilated, indicating biliary stasis, but the cultures of operative and postoperative bile, obtained in both operations, were always negative.

In another case, a 36-year-old female, the cystic duct was running along the medial aspect of the common duct down to the ampulla of Vater and was left long at cholecystectomy. Four years later, a single large cholesterol stone with a composition similar to that of stones removed at cholecystectomy was found. This patient had simple cholecystectomy at the first operation. Operative cholangiography showed no stone in the common duct or in the cystic stump at the cholecystectomy time. Bile culture, obtained at both operations, was always negative.

### *3. Patients with Monitoring of Operative and Postoperative Bile Bacteriology Through the T-tube (n = 131)*

Table 4 shows that patients with both intra and postoperative bile culture had a lower mean age (50.5 years, range 29–65) and less frequently a sphincterotomy (28.5%), than did individuals with a negative culture at operation, who subsequently had bile infection (mean age = 58.5 years, range 41–71; sphincterotomy associated in 88.8% of cases). Age difference between these two groups was statistically significant ($p<0.01$) as well as difference between the mean age of patients with both intra and postoperative negative culture and those with both intra and postoperative positive culture ($p<0.001$). Environmental bacteria were cultured in only 5 cases with a prolonged T-tube presence.

Therefore age, in association with sphincterotomy, was an important determinant of postoperative bacterial overgrowth in patients with previously negative cultures.

**Table 4** Monitoring of bile culture in patients with common duct stones and T-tube drainage (n = 131).

| *N* | *Mean age* | *Bile culture* *Intraoperative* | *Postoperative* |
|---|---|---|---|
| 39 | 50.5°* | – | – |
| 55 | 66* | + | + |
| 37 | 58.5° | – | + # |
| 2 | 68 | + | – |

: Associated sphincterotomy in 11 cases
: Associated sphincterotomy in 31 cases
### : 'Environmental' bacteria (through the T-tube) in 5 cases
*: p<0.001; °: p <0.01

### *4. Follow up Study of Patients with Previous Sphincterotomy or Sphincteroplasty (n = 105 of 287).*

Table 5 shows the data in the patients with postcholecystectomy stones that were found during the follow up study of the 105 patients (out of 287), who had radiological review. Thirteen patients with postcholecystectomy stones were detected. All underwent surgery and all had typical brown recurrent common duct stones. The mean time lapse after cholecystectomy was similar for both sphincterotomy and sphincteroplasty and was less than 4 years. The overall incidence (11.3%, i.e. 7 of 61 patients with sphincterotomy and 5 of 44 with sphincteroplasty) was also similar. In particular, no patient with non brown stones was found in this series.

**Table 5** Patients with recurrent stones after sphincterotomy and sphincteroplasty (n = 105 out of 287).

| | *N* | *Age* | *M* | *F* | *%* | *'t'* | *Morphology* | *stones composition* |
|---|---|---|---|---|---|---|---|---|
| SPHP | 5° | 64° | 1 | 4 | 11.1 | 3.8° | Brown pg. | CBR, CPL, CHL |
| SPHT | 7° | 68.8° | 1 | 6 | 11.3 | 3.4° | Brown pg. | CBR, CPL, CHL |

SPHT = Sphincterotomy; SPHP = Sphincteroplasty. 't' = mean time laps before recurrence (in years). CBR = Calcium bilirubinate; CPL = Calcium palmitate. CHL = cholesterol. pg = Pigment; ° = p:n.s.

### DISCUSSION

Sphincterotomy and sphincteroplasty are undoubtedly safe and effective surgical procedures[1–5]. In the 287 patients who were followed up, we observed no operative mortality and a very low perioperative morbidity (pancreatitis in 3 cases).

However, from present data we strongly suggest that:

(i) 84.3% of true recurrent common duct stones (i.e. brown recurrent plus non brown containing suture material) were brown pigment (27 of 32) (Table 1) and patients with brown recurrent common duct stones had a previous sphincterotomy in 70.3% of cases, and a biliary enteric anastomosis in 14% of cases (Table 2).

(ii) Postcholecystectomy stones in patients with cholesterol stones at the first operation were brown in patients with previous sphincterotomy and non brown if sphincterotomy was not performed ($p < 0.001$) (Tables 2 and 3).

(iii) The incidence of brown recurrent common duct stones in a follow-up study of patients with sphincterotomy or sphincteroplasty was 11% and no difference was observed in the incidence of recurrent common duct stones between the two procedures (Table 5).

(iv) Brown recurrent common duct stones may become symptomatic even after 12–27 years. Therefore, even if the mean time lapse for stone recurrence is usually shorter (3.5 years for sphincteroplasty and for sphincterotomy), it is evident that a longer follow-up period will detect an increasing number of patients with brown recurrent common duct stones.

After establishing that sphincterotomy and sphincteroplasty are associated with a recurrence rate higher than 11%, two questions require further comment:
1) What are the possible mechanisms that make sphincterotomy and sphincteroplasty able to produce brown stones? 2) Why do brown recurrent common duct stones form in some of the patients and do not in the others?

A definitive answer to these questions is not possible at the moment. Since bile infection is the crucial factor in the pathogenesis of brown recurrent common duct stones[7–15], probably the persistent alteration of the sphincteric mechanism, that acts as a protective system between the alimentary canal and bile tract, facilitates the penetration of Gram negative bacteria. After sphincterotomy, Gram negative bacteria enter the bile duct through the altered sphincter of Oddi. Data reported in

Table 4 support this view and are consistent with those reported by Gregg after endoscopic sphincterotomy[14].

However, stricture of a previous sphincterotomy or sphincteroplasty and bile stasis are probably required for bacterial persistance and proliferation.

Large bacterial concentrations, which could determine the precipitation of the typical components of brown recurrent common duct stones (calcium bilirubinate, calcium palmitate and organic matrix)[7–13], probably persist in the postoperative period only if a stricture is present.

Data in patients with biliary enteric anastomosis or with spontaneous enteric fistulas, showing that 80% of these patients have brown stones in the common duct and/or in the gallbladder (alone or in association with mixed stones), further support the view that the loss of the sphincteric function facilitates both bacterial overgrowth in the bile and brown stone formation[15]. Therefore the variable degree of sphincteric damage and the variable incidence of postoperative "stricture" (very difficult to define from a pathophysiologic point of view!) could explain, at least partly, why postoperative bactibilia does not lead to the recurrence of brown stones in every case.

Age of patients is also of importance. Table 4 shows that age, in association with sphincterotomy, is an important determinant of postoperative bactibilia in patients with previous negative culture.

In fact, it is well known that the duodenum is usually sterile in young healthy patients[10]. However, with the passing of time, immunological deficiency and motor disorders of the alimentary tract are known to increase[10]. In particular, bile culture performed in 1000 consecutive patients, as well as studies on the bacteriology of the duodenum and common duct bile in the same patients, confirm that bacterial colonization of the common duct is also an age dependent phenomenon[9,10,15,16].

Therefore, in young patients bactibilia is unlikely to occur after sphincterotomy (and therefore brown stones are unlikely to form).

However, when patients with a persistent damage of the sphincteric mechanism acquire with age those conditions that determine bacterial overgrowth in the duodenum, then bile infection is likely to occur and brown recurrent common duct stones can also form. In conclusion, the message is: "Avoid unnecessary sphincterotomy".

Endoscopic sphincterotomy has its own specific indications: acute suppurative cholangitis, stones impacted in the papilla, old patients or patients with repeated bile tract operations, palliation of bile duct tumors, or even in the diagnosis of a suspected ampulloma, etc.

Surgical sphincterotomy and sphincteroplasty should also be performed only when strictly required. Even strenuous supporters of sphincteroplasty report 6–10% of poor results in patients with a 5 year follow-up, controlled by i.v. cholangiography or ERCP [1–3]. The present study shows that at least 11% of patients with sphincterotomy are going to develop in the future brown recurrent common duct stones. Similar findings have also been suggested by others[17], even if this close relationship between sphincterotomy and recurrent common duct stones of the brown subtype has never been stressed specifically.

*Acknowledgement*

The present research has been supported by a grant from the Italian National Research Centre (CNR); Grant no 89.04131.04 and 89.02491.04

### References

1. Ribotta, G. and Procacciante, F. (1988) Transduodenal sphincteroplasty and exploration of the common bile duct. In: Blumgart L.H. (Ed.) *"Surgery of the liver and biliary tract"* pp.661–668. London: Churchill-Livingstone Publishers
2. Tondelli, P. and Schuppiser, J.P. (1988) Papillary stenosis. In Blumgart L.H. (Ed.) *"Surgery of the liver and biliary tract"*, pp.689–704. London: Churchill-Livingstone Publishers
3. Carr-Locke, D.L. (1988) Stones in the common bile duct. Endoscopic approaches. In Blumgart, L.H. (Ed.) *"Surgery of liver and biliary tract"*, pp.587–601. London: Churchill-Livingstone Publishers
4. Cotton, P.B. (1988) Preoperative endoscopic sphincterotomy as an adjunct to cholecystectomy for common bile duct stones. Hepatology Elsewhere. *Hepatology,* **8**, 191–192
5. Neoptolemos, J.P., Carr-Locke, D.L. and Fossard, D.P. (1988) Prospective randomized study of preoperative and endoscopic sphincterotomy versus surgery alone for common bile duct stones. *Br. Med. J.*, **294**, 470–474
6. Geeneen, J.E., Toouli, J., Hogan, W.J., Dodds, W.J., Stewart, E.T., Mavrelis, P., Riedel, D. and Venu, R. (1985) Endoscopic sphincterotomy: follow-up of evaluation of effects on the sphincter of Oddi. *Am. J. Surg.*, **149**, 668–671
7. Cetta, F. (1986) Bile infection documented as initial event in the pathogenesis of brown pigment biliary stones. *Hepatology,* **6**, 482–489
8. Cetta, F. (1983) Physicochemical and microbiological differences between primary recurrent and secondary common duct stones. *Chir. Epatobil.* **2**, 91–97 (in Italian, English abstract)
9. Cetta, F., De Nisi, A., De Mauro, D. and Alegente, G. (1983) Source of bacteria, bacteriology and antibiotic selection in biliary tract infection. *Chir. Epatobil.* **2**, 199–210 (in Italian, English abstract)
10. Cetta, F. (1983) The route of infection in patients with bactibilia. Letter to the editor. *World J. Surg.*, **7**, 562
11. Cetta, F., De Mauro, D., Bralia, A., Campopiano, M. and Petrini, C. (1984) Calcium palmitate biliary stones and their possible significance. *Gastroenterology*, **86**, 1314 (Abstract)
12. Cetta, F., Tommasini, E. and Saggese, N. (1985) Physicochemical analysis of pigment biliary stones. *Chir. Epatobil.*, **4**, 173–182
13. Cetta, F. (1985) Frequenza, composizione, struttura e patogenesi dei calcoli recidivi e residui della via biliare principale. Atti II Cong. SIPAD-AICEB Padova 31–40 (in Italian)
14. Gregg, J.A., de Girolami, P. and Carr-Locke, D.L. (1985) Effect of sphincteroplasty and endoscopic sphincterotomy on the bacteriologic characteristics of common bile duct. *Am. J. Surg.*, **149**, 666–671
15. Cetta, F. (1991) The role of bacteria in pigment gallstone disease. *Ann. Surg.* **213**, 315–326
16. Cetta, F., Frosini, G., Marini, M., Alegente, G., Marchi, B., Diele, G., Abdirahaman, A.H. and Petracci, M. (1989) Modificazioni con l'età ed il chimismo gastrico della microbiologia del succo duodenale. Atti XIV° Congr. Soc. It. Ric. Chir. Catania Monduzzi Ed. Bologna, 297–301 (in Italian)
17. Lygidakis, N.J. (1987) A prospective randomized study of recurrent choledocholithiasis. *Surg. Gynecol. Obstet.*, **155**, 679–684

*(Accepted by S. Bengmark 12 February 1991)*

